# An Overview of the Public Health Challenges in Diagnosing and Controlling Human Foodborne Pathogens

**DOI:** 10.3390/vaccines11040725

**Published:** 2023-03-24

**Authors:** Ayman Elbehiry, Adil Abalkhail, Eman Marzouk, Ahmed Elnadif Elmanssury, Abdulaziz M. Almuzaini, Hani Alfheeaid, Mohammed T. Alshahrani, Nasser Huraysh, Mai Ibrahem, Feras Alzaben, Farhan Alanazi, Mohammed Alzaben, Sulaiman Abdulaziz Anagreyyah, Abdulraheem Mousa Bayameen, Abdelmaged Draz, Akram Abu-Okail

**Affiliations:** 1 Department of Public Health, College of Public Health and Health Informatics, Qassim University, Al Bukayriyah 52741, Saudi Arabiae.marzouk@qu.edu.sa (E.M.);; 2 Department of Bacteriology, Mycology and Immunology, Faculty of Veterinary Medicine, University of Sadat City, Sadat City 32511, Egypt; 3 Department of Veterinary Medicine, College of Agriculture and Veterinary Medicine, Qassim University, Buraydah 52571, Saudi Arabia; 4 Department of Food Science and Human Nutrition, College of Agriculture and Veterinary Medicine, Qassim University, Buraydah 51452, Saudi Arabia; 5 Human Nutrition, School of Medicine, Nursing and Dentistry, College of Medical, Veterinary and Life Sciences, University of Glasgow, Glasgow G31 2ER, UK; 6 Department of Neurology, Prince Sultan Military Medical City, Riyadh 12233, Saudi Arabia; 7 Department of Family Medicine, King Fahad Armed Hospital, Jeddah 23311, Saudi Arabia; 8 Department of Public Health, College of Applied Medical Science, King Khalid University, Abha 61421, Saudi Arabia; mai@kku.edu.sa; 9 Department of Aquatic Animal Medicine and Management, Faculty of Veterinary Medicine, Cairo University, Cairo 12211, Egypt; 10 Department of Food Service, King Fahad Armed Hospital, Jeddah 23311, Saudi Arabia; 11 Supply Administration, Armed Forces Hospital, King Abdul Aziz Naval Base in Jubail, Jubail 35517, Saudi Arabia; 12 Department of Food Factories Inspection, Operation Sector, Saudi Food and Drug Authority, Riyadh 13513, Saudi Arabia; 13 Department of Preventive Medicine, King Fahad Armed Hospital, Jeddah 23311, Saudi Arabia

**Keywords:** foodborne pathogens, diagnostic approaches, control, phage therapy, public health

## Abstract

Pathogens found in food are believed to be the leading cause of foodborne illnesses; and they are considered a serious problem with global ramifications. During the last few decades, a lot of attention has been paid to determining the microorganisms that cause foodborne illnesses and developing new methods to identify them. Foodborne pathogen identification technologies have evolved rapidly over the last few decades, with the newer technologies focusing on immunoassays, genome-wide approaches, biosensors, and mass spectrometry as the primary methods of identification. Bacteriophages (phages), probiotics and prebiotics were known to have the ability to combat bacterial diseases since the turn of the 20th century. A primary focus of phage use was the development of medical therapies; however, its use quickly expanded to other applications in biotechnology and industry. A similar argument can be made with regards to the food safety industry, as diseases directly endanger the health of customers. Recently, a lot of attention has been paid to bacteriophages, probiotics and prebiotics most likely due to the exhaustion of traditional antibiotics. Reviewing a variety of current quick identification techniques is the purpose of this study. Using these techniques, we are able to quickly identify foodborne pathogenic bacteria, which forms the basis for future research advances. A review of recent studies on the use of phages, probiotics and prebiotics as a means of combating significant foodborne diseases is also presented. Furthermore, we discussed the advantages of using phages as well as the challenges they face, especially given their prevalent application in food safety.

## 1. Introduction

Foodborne infections have become more frequent over time and have now become a serious threat to public health globally, with more than 600 million people getting sick each year as a result [1,2]. Foodborne pathogens are responsible for thousands of infections and they are posing a risk to both the health of humans as well as the general economy due to their negative health effects [3]. A wide range of pathogenic microorganisms are capable of infecting food products throughout the manufacturing and processing processes as well as throughout storage and shipment processes prior to consumption [3,4,5]. The widespread intestinal disorders triggered by foodborne human pathogens are estimated to lead to a significant financial and health burden [6,7,8,9]. According to the World Health Organization (WHO), almost 30% of foodborne mortality is experienced by children younger than five years of age. Foodborne microorganisms may cause mild to severe symptoms, like diarrhea, or incapacitating diseases, such as meningitis [10,11,12]. This is because young children have weaker immune systems and are more vulnerable to foodborne illnesses. They can also be more likely to put their hands in their mouths or eat food that has been contaminated with bacteria, viruses, or parasites.

It is estimated that one out of four Americans falls ill every year due to foodborne infections, in spite of the fact that American food is among the healthiest food sources in the world [13,14,15]. A number of studies have found that the incidence and significance of different foodborne disorders is affected by the relationships among pathogenic organisms, human beings, foodstuffs and the environment [16,17,18,19]. As a result of foodborne illnesses, dangerous microorganisms such as bacteria, viruses, fungi and parasites are involved in the development of these diseases, although bacteria seem to be the most common cause and are capable of taking on a variety of characteristics and functions [3,20,21,22]. A number of bacteria, such as *Clostridium botulinum*, *Clostridium perfringens*, *Bacillus subtilis* and *Bacillus cereus*, can produce spores and are extremely heat resistant. As an example, *Staphylococcus aureus* and *Clostridium botulinum* are bacteria that are capable of producing toxins that are heat-tolerant [3]. In most cases, they are mesophilic, meaning their ideal growing temperature falls between 20 and 45 °C. Furthermore, certain pathogens, such as *Listeria monocytogenes* and *Yersinia enterocolitica*, that cause foodborne illness can survive in the refrigerator or at temperatures lower than 10 °C and can be transmitted to humans [23].

Among the numerous illnesses caused by foodborne microorganisms including botulism, dysentery, typhoid, gastroenteritis, listeriosis and a number of others, botulism is one of the most common [24]. A foodborne illness can be caused by any organism or its toxins, but it is most commonly diagnosed as acute gastroenteritis, and may present with other symptoms as well [25]. There is a wide range of severity and duration of symptoms associated with this illness. There is considerable reason to take foodborne pathogens seriously due to their zoonotic traits and their ability to produce toxins that cause illnesses or even death when exposed to them. Many countries and regions, both internal and external, are affected by foodborne pathogens in an increasing number of cases of occasional infections, ongoing problems, as well as massive and horrific outbreaks caused by bacteria that are present in food [26]. A large number of enteropathogenic bacteria are responsible for the large amount of diarrheal illnesses that occur every year among children under 3 years of age, resulting in more than 3 million deaths on a worldwide basis [27].

Many studies and reports [28,29,30,31,32] have found that intestinal cramping, diarrhea, vomiting, anxiety, chills and breathing difficulties are common signs of foodborne illnesses. These signs are caused either by consumed microorganisms, or toxic infections, such as *Clostridium perfringens* [33,34], or by microbial toxins produced by the microorganisms [35,36]. The concept of foodborne poisoning is based on the fact that it refers to illnesses caused by toxins produced by bacteria in food (for instance, signs caused by *Clostridium botulinum* food poisoning) [37,38]. Many of these infections are already attributed to specific foods, such as chicken, precooked meat, fish, dairy products, fruit and vegetables [39,40,41].

Clearly, it is of prime importance to identify foodborne pathogens in order to prevent them from causing significant harm to human and environmental health [42]. Culturing, biochemical analysis and immunoassay are the three most common conventional methods for determining foodborne pathogens [43,44]. However, a number of drawbacks can be found with these techniques, which include the time-consuming nature of performing these techniques, the length of the identification cycle, and the high costs and high level of operator skill required [45,46]. Consequently, there is a pressing need for the development of quick, cheap, accurate and easy-to-use methods for the identification of dangerous microorganisms in complicated food matrices so that they can be detected quickly and easily. Recent decades have witnessed an increase in modern technologies, which offers many benefits, including the ability to detect pathogens automatically, the ability to move and the ability to operate quickly [47,48]. A quick detection method is critical since it can identify pathogens in raw and processed foods, which is important for the food industry [49]. Using quick diagnostic techniques, small quantities of infections in food can be detected [50]. Having a high level of sensitivity is critical, since even a single pathogen present in food has the potential to cause human infection [22,51].

As part of this review, we examined a number of technologies that have been used to detect foodborne pathogens in recent decades, including culturing, immunoassays, genetic analysis, microfluidics, metabolic assays, biosensors, protein fingerprinting, and related methods. An overview of the premise, benefits and drawbacks of each method, as well as the current state of its application, is discussed in detail.

Attempts are being made by contemporary food production to decrease human illness by reducing microorganisms in food [52]. By maintaining proper monitoring, detecting infections in time, recording the origin, and removing contaminants in a timely manner on the farm, the risks posed by open-grazing animals, the environment and agricultural goods ingesting the food processing chain in the future can be greatly reduced [53,54]. In accordance with WHO, prerequisite programs are essential food safety measures prior to and during the implementation of hazard analysis and critical control point (HACCP) [55]. Among the global food safety community, HACCP is recognized as an important tool for reducing food-borne illnesses [56]. One of the most important steps for preventing foodborne infections is to ensure that the production line is run logically, high standards for cleanliness are implemented and biocides and sanitizers are used as frequently as possible [57]. However, there is a lot of inconsistency in the methods now used for the eradication of foodborne pathogens. Changing the product’s organoleptic characteristics can be achieved by steaming, heating, or using ultraviolet light [58]. There are also certain restrictions pertaining to the application of conventional antibiotic techniques, particularly those that pertain to fruits, vegetables, as well as ready-to-eat food, that may prevent the use of conventional antibiotics [58]. Using biocides on a regular basis promotes the development of microbial resistance, which poses a serious problem to public health [59].

The widespread overuse of antibiotics and the resulting growth of multidrug-resistant pathogenic bacteria has led to a paradigm shift in our understanding of antibiotics. Clinical failures associated with antibiotic therapy can be attributed to delays in the detection of foodborne pathogens [60]. Therefore, early detection of pathogens is essential to avoid the emergence of multidrug-resistant bacteria. Increasingly, concerns have been raised about the prevalence of antibiotic resistance among foodborne bacterial infections and its impact on treatment outcomes [61]. As multidrug resistance has become widespread in bacteria, alternative infection-control measures, such as prebiotics, probiotics and bacteriophages, are required to combat the problem [62]. A good example is the use of bacteriophages as biocontrol agents in controlling foodborne pathogens and preventing contamination of food contact surfaces [63]. A bacteriophage, or phage as it is sometimes known, is a virus that infects and multiplies in bacterial cells only if there is a host [64,65]. Due to their unique properties, phages are considered to be promising tools in the fight against bacterial diseases—as well as in the fight against many diseases—and have been widely used in modern medicine as a result [58]. Phage therapy is only one of the many applications for phages in industry. To ensure the safety of the consumer, every antibacterial treatment used in the food production industry must be carefully chosen before it can be used [58]. There are several advantages to using phages over chemical antibacterial agents, including the fact that they do not contribute to deterioration of the product’s quality; that they may be applied to a wide range of substrates; and that resistance to viruses can be addressed much more simply, in comparison to commonly used antibacterial agents [66,67,68]. It has been found that phages can naturally be found in food, which indicates that this is one way for them to come into contact with humans [69]. In addition to being cost-effective and easy to use, phage-based techniques are establishing themselves as an alternative to traditional antibacterial treatments.

Several studies have been conducted on the effectiveness of phages in eradicating foodborne pathogens [70,71,72,73]. It is of great importance to note that since phage research has grown significantly in the last couple of decades, we also discuss in this review the most recent discoveries on how effectively phages can be used against the principal food-related microorganisms and how these viruses may impact the method of microbiological management employed in food production. There is an imperative need to find effective alternatives that will allow the food industry to meet the high standards of food safety in the production of food items, as we believe maintaining the safety of food items to be a significant and major global challenge.

## 2. Pathogens That Cause Foodborne Illnesses

Pathogenic microbes, including bacteria, viruses, fungi and parasites, are responsible for causing foodborne illnesses [74,75]. Among foodborne pathogens, bacteria are the most common ones, causing a number of illnesses in both humans and animals [22,76,77]. In food, there are several common bacteria that are encountered such as *Salmonella* species, *Shigella* species, *Listeria monocytogens*, *Bacillus* species, *Yersinia* species, *Campylobacter* species, *Clostridium botulinum*, *Clostridium perfringens*, *Echerichia coli*, *Staphylococcus aureus* and *Vibrio cholera* [78]. A number of viruses have been identified as significant foodborne pathogens, which are capable of causing foodborne diseases [79]. These pathogens include hepatitis virus and norovirus (NoV) [76,80]. The presence of various parasitic species has also been found to pose a danger to the safety of food, including *Toxoplasma gondii* [81,82], *Giadia lamblia* [82], *Entamoeba histolytica* [82] and *Ascaris lumbricoids* [83]. Furthermore, there are a number of different species of fungi which may cause foodborne illnesses as well, including *Aspergillus flavus*, *Aspergillus ochraceus*, *Fusarium* species, *Penicillium patulum*, *Penicillium citrinum* and *Claviceps purpurea* [84,85].

## 3. Pathogenicity of Foodborne Pathogens

There are a number of virulence factors that bacteria use to penetrate into host cells in the complex process of microbial pathogenesis, in which the pathogen and the host are involved [86]. There are many virulence factors associated with bacteria, which include flagella, pili, fimbriae, adhesins (e.g., fibronectin, collagen, laminin, integrin and internalin) and biofilm formation (such as phospholipids, teichoic acids, nucleic acids, polysaccharides, proteins, capsules, enzymes, poisons and spikes) [87,88,89]. To combat the effects of infection, the host uses an array of immune cells such as phagocytic cells, complement pathways, stomach acids, mucus, bile salts and antimicrobial antagonists, as well as other mechanisms [90]. In case the bacteria are successful, infection is the final outcome. Nevertheless, humans may have been able to destroy or eliminate the pathogen if their immune systems were strong enough to destroy or eliminate it [91].

There are a variety of microorganisms which are found in food, such as viruses, bacteria, fungi and parasites, and which enter the mouth and travel to the intestines. The presence of foodborne microorganisms in foods may cause a wide range of diseases, ranging from localized infections to the spread of systemic infections capable of affecting practically every part of the human body [92,93]. To ensure foodborne infection occurs, a number of host-related factors must coincide for the process to take place due to the complexity of the process. Food or water that has been contaminated with the pathogen can cause the process to begin when a person consumes them [94]. The pathogen, in the long run, will potentially be able to infect a significant amount of host cells, causing significant damage. In the host cell, it remains persistent in the shifting environment, multiplies rapidly, and quickly spreads to different cells. To infect the intestine, the pathogen therefore makes use of chemotaxis, penetrating factors and sticky characteristics in order to get a hold of it [95]. Foodborne illnesses are mostly affecting people’s liver and gut, causing damage to those organs and resulting in liver damage and, in some cases, death [96].

## 4. Diagnostic Approaches of Foodborne Pathogens

The best way to prevent and control human foodborne illness is to continuously monitor and accurately identify the pathogens responsible for it [76,97]. By accurately identifying the pathogens that cause foodborne illness, food producers and distributors can take the necessary steps to ensure that their products are safe for consumption. This includes the implementation of safety protocols, such as proper storage and handling of food, to prevent contamination. It is increasingly accepted that traditional microbiological diagnostic approaches are too labor-intensive and time-consuming, particularly when it comes to identifying foodborne pathogens in order to meet the needs of rapid analysis of food samples [98,99]. As a consequence, new methods are needed in order to rapidly detect minute concentrations of live microorganisms within a given volume of food, which makes it very challenging to detect [99]. Recent advances in fast diagnosis and characterization of foodborne pathogens have been enabled by the use of nucleic acid-based techniques, immunologic methodologies and biosensor-based techniques [2,26,100]. As a result of false-negative or false-positive results, further testing may be necessary to determine the origin of these results [2]. Screening for food microbes has long been regarded as an essential step in the preparation of food, but it is typically used for reliable confirmation of the presence or absence of a given pathogen in a food sample.

**Figure 1:**
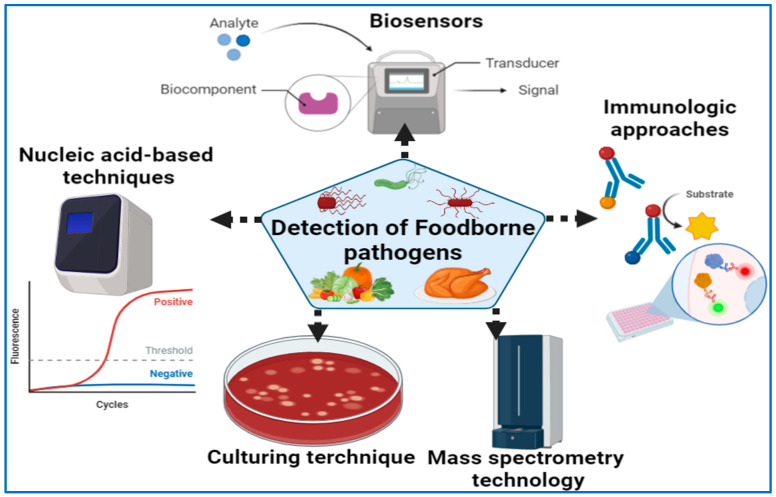
Detection of foodborne pathogens using various diagnostic approaches.

As far as microbiological analysis goes, there are two major purposes. One is to ensure that there are no pathogens or toxins in the food to ensure that it is safe to eat, while the other is to establish the total microbial load in order to determine product quality and shelf-life. The purpose of this section is to discuss a number of existing techniques, including culture-based techniques, matrix-assisted laser desorption ionization time-of-flight mass spectrometry (MALDI-TOF MS), polymerase chain reaction (PCR, and antibody-based immunoassays, which may be applied in the field (Figure 1). Culturing procedures, meanwhile, are based on the microbial growth that is used to identify particular pathogens [101,102,103]. The immunoassays are intended to measure the quantitative interaction of an antigen with its antibody [18].

### 4.1. Culture Techniques

In order to cultivate, isolate, count and develop the target microbe, the cultural methods use specific liquid or solid culture media, while simultaneously suppressing the growth of additional microbes present in the sample. During the process of determining foodborne pathogens, pre-enrichment growths, selective enrichment cultures and selective plating are used. Afterwards, serology and biochemistry are used to confirm the results. There is a wide range of approaches to understanding culture, both qualitatively and quantitatively [104]. Among the most fundamental microbiological techniques are the cultivation of bacterial and fungal cultures in vitro on nutritional media [105] because of their simplicity and straightforwardness of observation. In this approach, it is believed that food pathogens are identified by cultivating microorganisms on agar plates, followed by routine, specialized biochemical and serological tests for further identification of the organisms [106,107].

Agar media serve as a suitable medium for growing bacteria, which results in the formation of colonies when the bacteria grow in that medium. In terms of phylogenetic diversity, morphology and metabolism, fungi are more diverse than bacteria, and they are more culture-friendly than bacteria [107]. Bacteria and fungi that cause disease in humans are classified according to their morphology, which includes the size and form of their colonies, the color in the culture and the size and form of their cells, as well as their ability to reproduce. As a result of the pioneering work of Robert Koch, a number of criteria have been established that must be met for a bacterium to be labeled a disease-causing pathogen, a concept sometimes referred to as Koch’s postulates [108].

Several chromogenic/fluorescent media have been developed in order to replace traditional culture plating as well as to eliminate the need for subcultures and biochemical assays for microbial identification in order to replace traditional culture plating [109]. There are a wide variety of bacteria and fungi that are found in a variety of samples, so the lab workers would examine these samples and identify, isolate and distinguish particular bacterial or fungal diseases. Using chromogenic/fluorogenic media, it is only required to use a very small amount of material in order to identify harmful bacteria and molds. Whether it is culture broth or agar media, the presence of microbes can be detected through color production or fluorescence [110,111]. During chromogenic or fluorogenic substrate hydrolysis by a special enzyme produced by the microorganisms, a colored or luminous product is created, also known as color creation or fluorescence [112].

Because the activity of many enzymes is not species-specific, screening agents or enzymatic activity that is supplementary or secondary to the enzyme’s activity may be used to help determine the species of interest. In chromogenic media, selective agents (often antibiotics) are utilized to provide both positive as well as negative selection, as they are able to support the growth of target bacteria as well as inhibit the growth of non-target bacteria in the sample matrix [113]. For improved recognition and accuracy of bacterial selection and identification, it is possible to combine a number of distinct chromogenic enzymes and selective agents when using this method. A majority of the research on the chromogenic properties of enzymes is focused on the bacterial hydrolases (β-glucosidase or β-galactosidase in particular) [114,115]. As a result of the reaction between the chromogenic substrate and the bacterial enzyme, the substrates are given a vibrant color. The colonies are further differentiated by the presence of an enzyme that specifically recognizes the substrate.

According to the studies conducted by the Department of Disease Control in Paris, France, Rambach agar (CHROMagar, Paris, France) and the SM-ID medium were the first chromogenic media used to detect non-typhi salmonella strains in 1993 [116]. In 1994, a specially designed system called CHROMagar Candida was developed to distinguish and separate different Candida species. It is now widely accepted that there are a variety of commercialized chromogenic and fluorogenic media available on the market that can be used to detect pathogens in various samples based on the sample matrices used in various laboratories. As a matter of fact, the culture-based approach continues to be the standard method for determining the viability of pathogens; however, microorganisms’ ability to detect damage—or a state in which they are viable but not cultureable—can be affected substantially. Bacteria enter the non-culturable state when environmental conditions are negative, and these bacteria are unable to grow on regular nutrition medium due to their inability to survive [117].

It can also take a long time for bacteria to grow enough to form a visible colony and also for a chromogenic substrate to be added to a culturing solution, depending on (i) how fast a colony grows to form a visible colony, and (ii) how long it takes for an enzyme to produce color or fluorescence when the chromogenic substrate is added [118]. This method of culturing requires a lot of equipment to ensure that the cultures grow well and the results can be interpreted correctly. Other factors that can make it difficult to isolate pathogens in food samples are the low number of pathogens and the uneven distribution of them, the presence of native bacteria and the fact that food matrices can vary greatly. Microbiologists and clinical diagnosticians have focused more on culture-independent methods in the past few decades due to the sluggish and limited accuracy of microbe detection that has been experienced in food microbiology [119,120]. Despite their low cost, sensitivity and selectivity with chromogenic media-, culture- and colony-based methods are labor-intensive, time-consuming and may result in microbial contamination, which can inhibit growth of bacteria of interest and presence of viable but non-culturable bacteria.

### 4.2. Methods Based on Nucleic Acids

In order to identify specific gene sequences in an organism, these technologies utilize the genotyping of the target organism. Nucleotides, or sequences of nucleotides, can be used to identify groups, genera, species or strains of microorganisms that belong to a specific family or species [121,122]. A wide variety of DNA-based assays are available for detecting foodborne pathogens; however, nucleic acid amplification methods and probes seem to be the most popular and widely used methods for detecting foodborne diseases.

#### 4.2.1. Polymerase Chain Reaction (PCR)

There has been a long saga of molecular techniques developed in the last three decades, one of which is PCR [123]. It allows a quick replication of all, or a portion, of a unique DNA sequence within a cell or organism to enable further investigation of that particular genetic sequence. As PCR can only detect a single DNA sequence at a time, numerous PCR techniques are created and have been developed to detect the multiple pathogens present in a sample of food that is contaminated. Many of these techniques include multiplex PCR, nested PCR and reverse transcription PCR, as well as quantitative PCR to detect genetic information [124]. As multiplex PCR and quantitative PCR have become increasingly popular tools for detecting and diagnosing foodborne pathogens, our review aims to provide an overview of both techniques.

##### Multiplex PCR

A number of benefits are associated with multiplex PCR, including its ability to rapidly identify multiple species of harmful microorganisms, the high level of sensitivity and high specificity for the target organism [124,125,126]. By using this method, it is possible to quickly detect and magnify a wide range of bacteria in a single response, and multiple bacteria can be viewed at the same time. Using this approach, it would appear that the basic concept behind it is that a mixture of numerous primer pairs with varying target gene segments amplify the target gene simultaneously. There are several main applications of multiplex PCR, such as gene knockouts, mutation analyses and RNA detection. In doing so, it becomes possible to ensure that food is of high quality and safe to consume. In an example of multiplex PCR, Molina et al. [127] used multiplex PCR technique in order to identify *Escherichia coli* in food samples by detecting *lacZ* and *yaiO* genes. The multiplex PCR method was also successfully applied to identify *Listeria monocytogenes* in vegetables [128]. Using a multiplex PCR technique coupled with a membrane chip, Zhang et al. [124] developed a method that simultaneously detected nine types of foodborne pathogens. A number of other methods have also been created, such as the FilmArrayTM Gastrointestinal Panel (BioFire Diagnostics^®^, Salt Lake City, UT, USA) and the Gastrointestinal Pathogen Panel (GPP) created by Applied BioCode^®^ (Santa Fe Springs, CA, USA) to detect the 22 and 17 most commonly identified foodborne pathogens. With the aid of multiplex PCR, Li et al. [129] revealed the presence of three genes in *Vibrio parahaemolyticus*, which are named *tlh*, *tdh* and *trh*, respectively. There was a 19% prevalence of *Vibrio parahaemolyticus* in this investigation, indicating that this bacterium has the potential to cause serious health problems, as it grew and multiplied rapidly.

Consequently, multiplex PCR has proved to be an effective tool for the detection of a wide range of foodborne pathogens. However, to improve the efficiency of multiplex PCR analysis, it is important to determine if there is a correlation between the primers used in the different PCRs that might lead to poor amplification rates as the design of the primers plays a crucial role. A proper primer sequence should be constructed with a single annealing temperature in order to differentiate amplicons after a thermal cycle [130]. In addition, the inability to differentiate between alive and dead bacteria makes it possible to result in a false-positive result [131]. Therefore, multiplex PCR is frequently reported to produce subpar results because of its complexity.

##### Quantitative PCR

An integral part of the quantitative PCR reaction mechanism is the use of fluorescent reporter signals as a means of detecting PCR products. As a result of the presence of DNA that carries pathogenic properties, we are able to regularly determine whether the mixture contains pathogens by checking its fluorescence. Using a standard curve for calibration, it is possible to determine the quantity of pathogenic organisms present in a sample by measuring the degree of fluorescence within the sample [132]. Quantitative PCR is one of the most precise and selective methods of genetic testing. There is no need for post-PCR DNA analysis since real-time detection of PCR products eliminates that requirement. It can be argued that due to these factors, the quantitative PCR technique has become one of the most commonly used methods for identifying and classifying foodborne pathogens for its accuracy and effectiveness. As a result of the rapid development of TaqManTM and LightCycleTM probes, quantitative PCR procedures are becoming more frequent [124]. It has previously been demonstrated that quantitative PCR has been used to identify three subtypes of the *stx1* and seven subtypes of the *stx2* associated with *Shigella* toxins [133]. Similarly, Nadin-Davis et al. [134] used the quantitative PCR method for analyzing samples collected from 239 poultry operations as part of their study to identify Salmonella in the samples.

A quadruple probe quantitative PCR method has been developed by Alia et al. [135] in order to determine whether there were different serovars of *Listeria monocytogenes* present in packaged and processed meats. There is a high level of precision, speed and sensitivity associated with the probes. Their study findings were crucial for identifying if there was a risk of *Listeria monocytogenes* contamination in meat products as well as how to establish routine monitoring of the presence of persistent strains of this organism in ready-to-eat meat products as a precaution. According to Zhang et al. [124], a nationwide standard procedure in combination with quantitative PCR techniques was used to systematically identify 60 animals, marine and dairy products in order to determine their susceptibility to foodborne illness. Quantitative PCR technology actually showed a higher rate of pathogen detection than the national standard approach, even though *Staphylococcus aureus*, an essential pathogen, was missed.

The presence of *Salmonella* in grilled ham was reported by Martin et al. [136]. According to the authors, it appears that the limit of detection was set at 10^3^ CFU/g for this organism. There were three organisms identified in the analysis of fresh pork by Ma et al. [137]: *Staphylococcus aureus*, *Salmonella* and *Shigella*. Their detection time was less than 8 h, which is significantly shorter than that of standard PCR detection times. According to the study’s findings, *Shigella* had a detection limit of 6.8 CFU/g, *Salmonella* 2.0 CFU/g, and *Staphylococcus aureus* 9.6 CFU/g. An increase in the detection efficiency of *Escherichia coli* O157:H7 was observed by Ranjba et al. [138], and the species of this organism is now known to be easy to detect. In some cases, the detection might only take a couple of minutes to complete. According to the findings of the study, the detection limit for this strain was 78 pg/tube.

Standard culture techniques, along with quantitative PCR, yielded reliable results, but by using quantitative PCR, the results were obtained more quickly. Because the quantitative PCR technology uses a closed system for preparing the product, false positives from contaminants at the moment of analysis can be avoided. Quantitative PCRs are extremely quick since they are performed on a standalone instrument without any need for electrophoresis, resulting in a significant reduction in the total time of the process compared to conventional PCR, which require electrophoresis after the amplification is complete. The real challenge with multiple probe Quantitative PCR is that it is challenging to perform as the operator needs a high level of technical skill, the equipment is expensive and the technology itself is expensive [139,140].

#### 4.2.2. Isothermal Amplification

The procedure known as isothermal amplification of nucleic acids can be described as a simple, rapid and effective way to accumulate nucleic acid sequences under fixed thermodynamic conditions. Since the early 1990s, several isothermal amplification techniques have been developed as alternatives to PCR, which is an effective DNA amplification technique [141]. These isothermal amplification techniques are widely used in biosensing systems and can be used to detect DNA, RNA, cells, proteins, tiny molecules and ions, as well as other biological targets. In recent years, isothermal amplification has gained popularity as an effective strategy for identifying foodborne pathogens. The appeal of this method is that it does not require specialized and expensive equipment which is why it is very appealing to many companies involved in food production. It is generally regarded that there are two basic techniques of isothermal amplification, loop-mediated isothermal amplification (LAMP) and nucleic acid sequence-based amplification (NASBA) [142].

##### Loop-Mediated Isothermal Amplification (LAMP)

**Figure 2:**
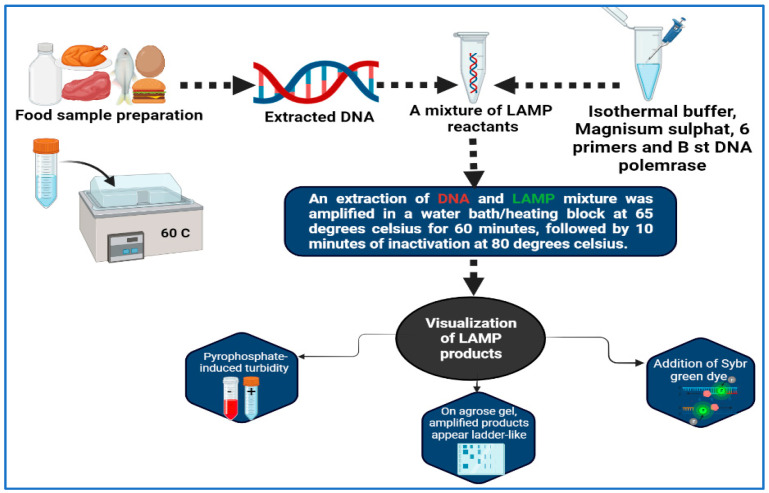
Workflow of LAMP for detection of foodborne pathogens.

Molecular amplification of nucleic acids is a technique that has been developed by Notomi et al. [143], who developed the LAMP method. The LAMP technique is very specific, since it uses four or six different primers and binds one from each pair to six or eight (sometimes) specific locations on the target gene itself. All of the reactions can be conducted at isothermal conditions between 60 and 65 °C, which is the ideal temperature range (Figure 2). A post-amplification electrophoresis is not needed for the detection of PCR products since the process can be carried out visually without the use of any device [144,145]. First of all, the detection of *StxA2* by LAMP is a first in the area of foodborne pathogen detection, having been performed in *Escherichia coli* O157:H7 strains [146]. It can possibly be argued that the characteristics of LAMP are less hazardous to health than those obtained by in situ PCR due to the mild permeable nature and low isothermal temperature used. According to the results, this approach is more successful in producing images that feature more contrast than the in situ PCR method [147]. The LAMP method was used by Hassan et al. [148] for the purpose of determining the frequency of foodborne pathogens associated with dairy products. In less than 30 min, a single copy of a gene can be identified using LAMP, thus making it extremely quick and sensitive. The LAMP technique has been designed and evaluated by Zhao et al. [149] as a method for identifying foodborne *Salmonella* strains rapidly. An optimal reaction temperature of 65 °C for 45 min was found to be optimal when detecting 1 pg of DNA per tube and 100 CFU per reaction. A total of 214 *Salmonella* strains isolated from food sources were subjected to rapid and simple LAMP screenings. For both LAMP and PCR, the accuracy rates were 97.7% (209/214) and 91.6% (196/214), respectively, with 100% specificity. Based on the results of 39 reference strains that were tested simultaneously using the LAMP assay, high specificity was achieved and no false positives occurred.

As a result, it can be concluded that the LAMP test is a convenient and effective method of identifying *Salmonella* strains in a timely manner. This modified LAMP approach has advantages in terms of simplicity and time consumption compared to previously published LAMP analyses used to identify Salmonella strains. A total of 60 min was spent on the entire detection process, including DNA extraction, LAMP reactivity, reporting the results and interpreting the data. In all probability, LAMP assays will be widely used for the identification of microbiological *Salmonella* species based on their speed, simplicity and affordability. As far as identifying particular infections is concerned, LAMP is one of the easiest methods. An amplification reaction can be completed at a steady temperature, as long as the temperature remains constant. This technique has been shown to be far more sensitive than traditional PCR because of the advantage in sensitivity that LAMP offers. There is no doubt that one can complete the test in 30–60 min [150,151]. Unless there are measures taken to prevent cross-contamination, an amplified sequence has a maximum length of 300 bp; furthermore, non-specific pairing between loop primers will yield false positive results and cross-contamination can occur unless precautions are taken to prevent it [124]. There are also a number of complications and costs involved in the creation of primers.

##### Nucleic Acid Sequence-Based Amplification (NASBA)

In contrast to conventional PCR, which is used to detect DNA, NASBA uses isothermal amplification of RNA from the target organism, making it more sensitive than conventional PCR [152]. In order to perform thermal cycling, there is no need for any additional equipment. As soon as NASBA detects the targeted sequence of RNA, a primer is attached to it, and then reverse transcriptase creates a strand of cDNA from the primer. A second primer for reverse transcription is attached to the template RNA, after which the template RNA is digested by RNase H, to create double-stranded cDNA by reverse transcription of the template RNA. In the end, RNA transcripts are generated through amplification using T7RNA polymerase in the presence of amplification product [153]. Since RNA is amplified using an RNA polymerase without being converted to cDNA, this technique is exceptionally well-suited for testing for RNA viruses due to the fact that it uses an RNA polymerase without being converted to cDNA [154,155]. The NASBA analytical approach has been successful in detecting a number of foodborne pathogens that have been sensitive, specific and fast enough to be applied in a number of food-borne situations [18,156].

However, NASBA is not making any progress toward being adopted as an analytical method in food analysis, despite its well-established status as a clinical diagnostic method with significantly greater specificity for pathogen detection compared to quantitative PCR. Despite the limitation, this is a real disappointment, because it is different to PCR in that it can detect live cells by amplification of messenger RNA, even if genomic DNA is present in the sample. Although the NASBA method was quite reliable, it occasionally revealed the presence of amplifications that were not expected. It is imperative to develop techniques that can be used effectively to detect live microbial pathogens in food quickly, accurately and specifically, and furthermore, it may be worth exploring and utilizing the potential of the NASBA in this regard [157].

#### 4.2.3. Diagnostic Immunology

Since the advent of immunologic diagnostic techniques and their widespread use, immunological diagnostic tools have evolved tremendously from their initial invention into the present day; they have established a hegemonic status in the fields of biological science, food science, and clinical medicine. In immunological detection, specific antibodies (polyclonal antibodies or monoclonal antibodies) bind to specific antigens, which is known as an antibody–antigen interaction [158,159,160,161]. The detection of microbial toxins and pathogens in food has been accomplished with the use of several antibodies. Specificity of the antibodies is the most important factor in determining the appropriateness of the antigen–antibody combination [146]. A polyclonal antibody, which is made from either rabbit or goat serum, contains multiple antibodies of varying biological origins and levels of specificity. When identifying molecules with precision, monoclonal antibodies are frequently more helpful than polyclonal antibodies. Immunological detection of microbial contamination has become more specific, sensitive, reproducible and reliable with the development of monoclonal antibodies [162]. There are two immunologically based techniques that are being used increasingly for the detection of foodborne microorganisms: lateral flow immunoassays and enzyme-linked immunosorbent assays (ELISA).

##### Enzyme-Linked Immunosorbent Assay (ELISA)

In terms of a diagnostic biochemical test, ELISA can be regarded as a sensitive and specific method that does not require the use of elaborate or expensive instruments for the detection, quantification and analysis of analytes [163,164,165]. Although conventional ELISA is widely utilized in scientific research and testing organizations today and is regarded as an effective technique for the identification of viruses and antibodies, this conventional method requires skilled operation techniques and it is also extremely time-consuming [166,167]. Therefore, the researchers modified the conventional methodology in order to carry out their study. A variety of anti-NoV antibodies were tested by Zuo et al. [168] using an indirect ELISA, which they developed to evaluate the antibodies. Throughout the entire testing process, two hours were needed to complete the testing of the entire range of unknown antibodies. Within and between the tests, there was no difference in the coefficients of variance, which both fell under 10%. In a sandwich-ELISA based on the *Salmonella enteritidis* antigen, He et al. [169] successfully found 6 CFU/mL of *Salmonella enteritidis* in milk after 10 h of enrichment. A wax-printed paper-based enzyme-linked immunosorbent assay based on microfluidic paper-based analytical equipment was developed by Zhao et al. [170] in less than 3 hours and a sample volume of only 5 μL was required for detection. In the case of *Echerichia coli* O157:H7, the limit of detection was 10^4^ CFU/mL. There are many advantages of ELISA tests, including simplicity, high specificity and sensitivity, high efficiency (since multiple tests can be conducted simultaneously), and cost-effectiveness (because reagents are cheap) [171]. However, it is labor-intensive and expensive to make antibodies, and expensive growth mediums are necessary to obtain a specific antibody. Moreover, there is a risk of false positives or negatives due to insufficient blocking of the antigen-immobilized microtiter plate surface [171].

##### Immunomagnetic Separation Technology (IMS)

As one sample preparation technique of pre-concentrating food samples, IMS has been proposed to rapidly and precisely remove and concentrate target germs from challenging food substrates [172,173]. Generally, superparamagnetic particles are chemically modified, and then they are combined with antibacterial proteins made from immunomagnetic beads separation, which then can be used to identify and capture the target bacteria in the samples that are being tested according to the main theory [174,175]. This process allows the antibodies on the immunomagnetic beads separation to identify and capture specific bacteria. In order to obtain the precise and effective concentration of the microorganism, a magnetic field is used to quickly separate the complex from other contaminants present in the sample so that it can be separated effectively and precisely [176,177]. The IMS technology has been demonstrated to have the following qualities: high sensitivity, excellent specificity and a rapid separation speed [178]. Among the disadvantages of IMS approaches is the difficulty of isolating complex phenotypes [179]. Immunomagnetic separation and fluorescence-activated cell sorting both have known or unknown drawbacks. Because the target cells are likely to absorb associated components (e.g., antibody–fluorescence complexes, antibody–magnetic beads), their phenotypes or viability might change [180]. In order to improve the effectiveness of the detection process, it is possible to combine it with other technologies such as ELISA, chemiluminescence immunoassays, flow cytometry, PCR and other detection methods [181,182].

#### 4.2.4. Mass Spectrometry Technology

An unbiased and accurate method to detect pathogenic bacteria is mass spectrometry (MS). Since the advent of mass spectrometry, this method has steadily evolved into a new way to detect various types of pathogenic bacteria [22,51,102,183,184]. A non-biochemical instrumental analytical technique used to examine the characteristics of bacteria or a portion of their proteins as the subject matter of research, MS refers to the technique of analyzing the ions produced after ionization in order to identify and detect the target bacteria. A new method has been developed by Feucherolles et al. for detecting antimicrobial resistance in bacteria. For relevant foodborne pathogens such as *Campylobacter coli* and *Campylobacter jejuni*, a combination of protein mass spectra from matrix-assisted laser desorption/ionization time-of-flight mass spectrometry (MALDI-TOF MS) and a prediction method was developed [185]. Furthermore, Li et al. [186] have developed a unique method for MALDI-TOF MS simultaneously detecting numerous bacteria by using mass tags induced surface engineering in conjunction with a MALDI-TOF MS procedure in the same study.

As a result of this method, mass tags are no longer only used to identify pathogens, but can also be used to identify multiple bacteria at once without the need to collect microbial mass spectrum libraries before analysis. According to Dias et al. [187], they examined the antimicrobial properties of three essential oils and their necessary ingredients against foodborne pathogens and spoiled food. They indicated that the gas chromatography-mass spectrometry and gas chromatography-flame ionization detector methods were used. MS is currently the most effective detection method for foodborne pathogenic bacteria, providing quick detection accuracy and ease of operation, but there have been many issues with the actual identification process [188]. It is necessary to continuously troubleshoot spray voltage, flow rate, capillary temperature, and other difficulties during the detection process as part of the process to increase the sensitivity and reliability of foodborne pathogenic bacteria detection technology. MALDI-TOF MS has several limitations, such as an inability to distinguish between related species due to their inherent similarity. In addition, misidentification may occur when some members of a species complex are listed in the database, but others are not [189].

#### 4.2.5. Detection Technology Based on Biosensors

Biosensors are used as a tool for analysis that consists of two components: the biosensor and the transducer. Microbes can be identified primarily by their antigens (antibodies), sensitive enzymes, alkaloids and their gene sequences [190,191,192]. The materials being tested undergo biological reactions when they are in contact with these substances. By using signal transducers, these interactions can be converted into quantifiable electrical signals, which are then detected and read by amplifiers [193,194]. A variety of types of biosensors can be classified as biosensors based on their operating principles, including optical, electrochemical, enzyme, physical and mechanical biosensors [195,196,197,198]. There are two types of biosensors commonly used for the rapid detection of foodborne pathogens: optical biosensors and electrochemical biosensors [199,200]. The rapid detection ability of optical biosensors, as well as their sensitivity and selectivity, make them extremely useful for identifying foodborne pathogens [201]. The most popular optical sensing technologies currently being used are chemiluminescence, colorimetry, fluorescence and surface plasmon resonance [202]. Quintela et al. [203] used silver nanoparticles that were functionalized with oligonucleotide for optical biosensing. With an effective pooling method and a complex matrix that ensured viable cells detection, they developed a novel method for simultaneously detecting *Salmonella* species strains optically in complex matrices. Based on the results of the study, it was confirmed that the test was able to detect <10 CFU/mL of the target bacteria with a 100% specificity, thus making it highly sensitive to its target species. Using an electric control-based photocatalytic nanocomposite in conjunction with a nanophotonic structure, Srivastava et al. [204] were able to detect foodborne pathogens without a label by using optical nanophotonic detection.

Biosensors are capable of identifying *Escherichia coli* at a concentration of 5000 CFU/mL, which is very high. In order to detect *Salmonella typhimurium* lipopolysaccharide and *Salmonella* bacteria in drinking water, Angelopoulou et al. [205] used white light reflectance spectroscopy to develop an optical biosensor. The experiment took approximately 15 min to complete, and the detection limits for bacteria and lipopolysaccharide were determined to be 4 ng/mL and 320 CFU/mL, respectively. It has been shown that electrochemical biosensors undergo electrochemical reactions with target analytes on the electrode interface, resulting in changes in currents, potentials, inductances or permeability on the sensor surface [206,207]. A biosensor is an electrochemical system that uses an electrode as a signal converter. There are three different functional porous pseudo-carbon paste electrodes that can be fabricated [208]. There is a linear correlation between the peak current of porous pseudo-carbon paste electrodes and the *Escherichia coli* O157:H7 concentration between 1.0 × 10^3^ and 1.0 × 10^7^ cells /mL, with a limit of detection as low as 8.0 × 10^2^ cells /mL when the porous pseudo-carbon paste electrode was used as the anodic stripping anode. The target analyte can be measured by keeping an eye on the fluctuations in these signals while they are being analyzed. As a method for developing a label-free and quick electrochemical biosensor for *Listeria monocytogenes* detection, Oliveira et al. [209] converted platinum and chitosan into a biomimetic nanostructure that responds to pH variations. 

Using a reduced graphene oxide-gold nanocomposite, Jia et al. [210] developed an electrochemical sensor for detecting *Staphylocccus aureus* using single-stranded DNA as an aptamer. By using ferri-/ferrocyanide as a redox probe, Bekir et al. [211] identified *Staphylococcus aureus* using impedance spectroscopy by monitoring the change in resistance before and after *Staphylocccus aureus* was immobilized on a gold electrode. As a result, the biosensor was utilized to detect pathogens that had been stressed and resuscitated. According to Jiang et al. [212], electrochemiluminescence can be used to detect DNA from Clostridium perfringens via rolling circle amplification, as reported by Zhao et al. [213]. An electrode-based Clostridium perfringens DNA biosensor was published by the same research group in the same year [212]. The biosensor technology detects microorganisms at a high rate and with a high degree of sensitivity; moreover, the operation is simple and the personnel requirements are low [214].

#### 4.2.6. Next-Generation Sequencing (NGS)

Introducing next-generation sequencing (NGS) methodologies may make detecting foodborne infections more accurate than the methods currently in use [215]. A major advantage of NGS is its ability to detect numerous potential agents of spoilage and disease, as well as identify species that are both culturable and non-culturable, without having to do any targeted analysis. Calo-Mata et al. [216] demonstrated the potential of using liquid chromatography-mass spectrometry to detect non-targeted bacterial species. However, lack of standardization and lack of public databases restricts their use. Also, unlike NGS, these approaches cannot provide information on the strains and genomes of bacteria [217]. Food microbiology investigations are increasingly utilizing NGS, with most of them focusing on whole genome sequencing (WGS) of bacteria isolates and discovering microbiomes associated with certain commodities [218,219].

Several previous publications have discussed the benefits of using metagenomics to assess the food microbiome, such as their ability to identify multiple pathogens simultaneously. In addition, multiple pathovars of the same pathogen are being identified as well as identification of genes of interest, including those associated with antibiotic resistance [220,221]. NGS technologies, however, have inherent limitations. They are computationally expensive, produce short read lengths and generate errors. Assembly software cannot resolve large structural rearrangements (insertions, deletions, inversions) in de novo sequencing and disambiguate repeat regions [222].

## 5. Antimicrobial Resistance and Food Safety

There are numerous steps in the food production process that contribute to the prevention and control of food-borne illnesses, including transporting, storing, processing and preparing food, as well as assuring the safety and health of food for humans [223]. Food-borne illness is one of the most serious public health threats associated with antibiotic resistance. Previous studies have indicated that an increase in antibiotic-resistant bacteria has led to an increase in foodborne illnesses [224]. In recent years, numerous studies have been conducted to find solutions to antibiotic resistance, especially resistance spread from food to humans [224]. There are a number of antibiotic-resistant bacteria in foods and humans; however, some basic and easy-to-follow steps can help prevent the spread of antibacterial resistance foodborne pathogens, like appropriate hand washing, convenient vegetable washing and cooking temperatures. Mensah et al. [225] state that antibiotic residues can negatively affect public health in a number of ways, such as nephropathy, allergic reactions, hepatotoxic effects and carcinogenicity.

There are four main consequences of antibiotic-resistant bacteria and infectious diseases: (1) delay or failure in treatment; (2) restrictions in antimicrobial selection; (3) survival of resistant strains in other bacterial illnesses; and (4) coexistence and increased pathogenicity of resistance genes due to selection [226,227]. Studies conducted in the past have indicated that numerous food-borne pathogens are resistant to a variety of antibiotics. Some examples of significant resistant foodborne bacteria include Thermotolerant *Campylobacter*, *Salmonella*, *Staphylococcus aureus* and *Enterococci* species [224].

## 6. Control of Foodborne Microbial Pathogens

### 6.1. The Role of Bacteriophages in Combating Foodborne Pathogens

A bacteriophage, or phage as it is sometimes called, is a virus that attacks and multiplies only in bacteria and is linked to bacterial infections [228,229]. Among the many industrial applications of phages, phage therapy is one of the most promising ways for them to fight bacterial diseases as a result of their unique characteristics that show they can be effective in combating bacterial diseases [58,230]. With the aim of ensuring the safety of consumers, every antibacterial treatment used in the food production industry must be carefully chosen because each treatment used should aim to ensure the health of consumers. In contrast to commonly used antibacterial methods, phage treatment does not affect the quality of the product in any way, and viruses may be applied to a different range of matrices, which provides a much simpler approach than the use of chemical antimicrobials in order to overcome resistance problems [66,67,68]. Several phages have been identified in a range of foods, which indicates that this is one of the ways they can naturally get into the bodies of humans [69,231]. Because phage-based approaches include all of the advantages of traditional antibacterial techniques, such as affordability and ease of use, there is no reason not to use them in the future.

According to several research studies [70,71,232], phages may be an effective means for eradicating foodborne pathogens that cause food poisoning. This study discusses the most recent discoveries about phages in relation to food-related pathogens. Since phage research has seen a dramatic increase in the last few years, we present a summary of the findings of this study. We also discuss the different ways in which these viruses can be utilized to manage food production in biologically efficient ways. As a matter of fact, we believe that protecting food items from contamination is one of the greatest challenges facing the world, and that it is urgent for us to address this challenge by searching for new solutions that allow for the maintenance of high criteria for food security. There is no doubt that maintaining the protection of food items is one of the major challenges we face on a global scale, and we must look for novel solutions that will be able to maintain the requirements for food safety that are as high as possible.

**Figure 3:**
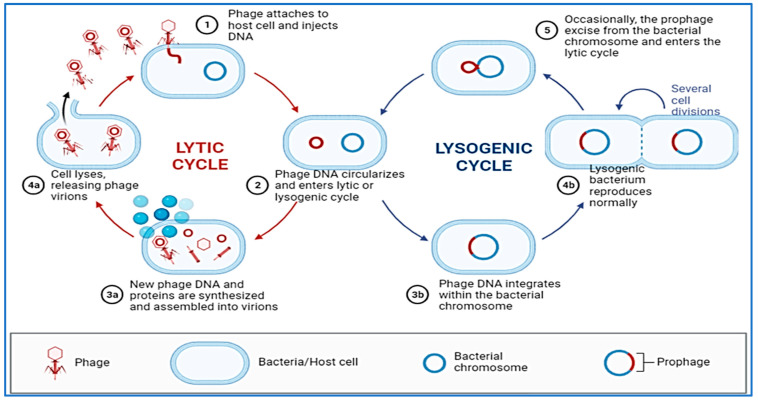
Replication process of bacteriophages (Lytic and Lysogenic cycles).

Compared to other types of microorganisms, bacteriophages have a high degree of selectivity when it comes to which species or strains of bacteria they are able to infect [233,234]. As a result of their selective interactions at the surface of the bacterial cell, the viral receptor and its ligand are able to achieve this high degree of specificity. As an entry point for bacterial cells, phages may utilize proteins that make up the outer membrane, pili, flagella, polysaccharides, lipopolysaccharides and amino acid moieties to let them in. There are two main cycles in the replication process of bacteriophages (Figure 3). When the phage’s genetic material enters the proper host, it is duplicated and copied using the bacterial molecular machinery in the process of lytic cycling, which occurs during the initial stages of the phage’s lifespan. Soon after the phage penetrates into the cells of a host organism, new virions are generated and released into the environment. Due to this chain of events of molecular occurrences that have been linked together, the bacterial cell lyses [235]. Lytic phages are phages that are capable of multiplying through the lytic cycle, and are therefore called virulent phages [236]. This virus replicates with its host as a prophage through the lysogenic cycle, an important part of which is the combination of the nucleic acid of the virus with the chromosome of the host. A change in the environment, which can be unfavorable to the phage, could nevertheless cause it to switch to the lytic cycle [237]. Because of the lack of lytic activity, temperate phages cannot be used for practical purposes because they are not suitable candidates to be used as phages in practical applications.

Phage therapy proponents point out that phages have a variety of important benefits over antibiotics, including host-specificity, self-amplification, biofilm destruction and low toxicity to humans. The presence of several characteristics of bacterial viruses, in contrast with conventional chemical antibacterial agents, has come to be seen as an advantage in the years after the deployment of phages and has been viewed as advantageous in several circumstances. As bacteriophages exclusively detect and infect only a specific type of bacterial host, they do not interfere in any way with the microflora of the organism. The human body can cope better with these types of therapy than antimicrobials, which is what makes them a safer form of therapy [229,238]. There has been some evidence that bacteriophage products can achieve their desired beneficial effect with one dose of the bacteriophage product [239,240]. Despite the fact that many phages have the ability to multiply where they find their hosts, the key to this process is the “auto-dosing” characteristic, which is one of the unique features of phages [241]. Because they live within our body, bacteriophages are found in areas of the body such as our gastrointestinal tract or respiratory tract. It has been reported that the first viruses enter the intestines within four days after the birth of an infant [229]. The unintentional exposure to phages will allow us to confirm the safety of the intended phage application. A significant aspect of phage recruitment is the ease at which it can be done, and the fact that it is faster and cheaper than the acquisition of antimicrobials [242]. The phages used in food safety applications should be sufficiently sterile to avoid affecting the organoleptic, rheological, or nutritional characteristics of the food products [243].

Phage use is limited by other restrictions, such as their limited range of activity, which may prohibit the development of universal preparations or the emergence of phage-resistant strains. In addition, the use of phage cocktails, which are made up of phages with a wide range of specificities, can broaden the scope of lytic activities of a preparation, or phages with comparable activities, can be used to overcome both of these limitations [242,244]. The preparation may contain phages that will be susceptible to bacteria even if they develop resistance to one phage from it, but they may still be susceptible to another [242,244]. Taking into account other factors, such as virulence enhancement, antibiotic resistance genes activation, or phage coinfections, it is imperative to emphasize that phage cocktail efficacy is complex, as there are many different factors that must be taken into account [245].

As a result of global concerns about consumer food safety, evaluating the techniques used by households to process food has been the subject of extensive research. In order to reduce the incidence of foodborne illness, consumers and food safety educators must be aware of activities that can prevent exposure to foodborne pathogens. The development and implementation of food safety education programs are necessary for the improvement of food safety behaviors [246]. A wide range of antibacterial tactics are being used today, all of which have their own drawbacks, which is why it is highly desirable to develop alternatives which are simple to use, do not affect product quality and are secure and inexpensive. Several studies have shown that bacteriophages have high efficacy against the most common pathogens of foodborne origin, and numerous scientists have expressed their keen interest in leveraging the advantages of bacterial viruses when it comes to control of these pathogens [62,247,248]. In this context, it should be noted that the research in this area is much more developed compared to other sectors of the food industry, which is why we are focusing only on reviews of reports published on the efficacy of bacteriophages in eradicating food-related microorganisms in this review.

Despite the fact that bacteriophages are useful antibacterial agents that have numerous advantages over other antibacterial agents, they also have a number of disadvantages, particularly when phages are used extensively. The phage’s ability to transduce bacterial genetic material is well-known—which of course, is the ability for phages to transfer pieces of bacterial genetic material arbitrarily across the cell membrane. During transduction, it is possible that virulence or antimicrobial resistance genes will unavoidably be transferred into an organism. These genes could have a serious adverse effect on the human body, spreading dangerous strains of bacteria into the environment and putting humans at risk [61]. A number of factors must be taken into account when making use of bacteriophages so that the potential for harm is minimized. Moreover, no comprehensive study has been carried out on the direct impact of phages on the human body, and the direct impact on the human body is yet unknown. It is interesting to note that the assumption about the safety of using phages is mostly based on clinical experience and not on scientific understanding, which can be evident from many years of clinical experience rather than a scientific understanding. According to studies conducted over the past few years, bacterial viruses have been shown to interact with mammalian cells [249,250,251]. As a result, such reciprocal interactions deserve careful consideration.

### 6.2. Probiotics

There is compelling evidence that the probiotics that are living bacteria and yeasts carry positive effects for humans as well as animals [252,253]. It has been emphasized by WHO and FAO that probiotics reflect the position of living organisms which, when taken in appropriate amounts, can exert a beneficial effect upon the body. Probiotics are among the most commonly distributed species in the intestine of both humans and animals. The majority of them enhance the intestinal microbiota equilibrium of the host animal or alter the characteristics of the native microflora in the intestine of the host [254,255]. Due to the growing number of diseases and the aging of our population, it is becoming more and more important to apply knowledge about the microbiota of the digestive tract and the positive effects of probiotic bacteria in the digestive tract in an effort to combat these diseases [256]. The ingestion of preprocessed meals (fast food) is one of the factors that adversely affect the human gut microbiome, as they regularly contain enormous quantities of fat and inadequate amounts of vegetables, which are both factors that negatively affect the gut microbiome [256]. As far as we are concerned, there is now no doubt that gut microbial populations are highly significant to overall health and protection. That is why probiotic formulations and products can increase the number and diversity of gut bacteria as well as protect individuals from gastroenteritis [256].

Human health can be greatly improved by probiotics because of their wide range of beneficial effects. There are several advantages to these agents, which primarily relate to their impact on the growth of the microbiota that lives inside the organism, thereby ensuring the dynamic balance between bacteria and pathogens that is necessary for the organism to function normally [257,258]. These agents are able to act in a preventive manner to prevent the proliferation of pathogenic bacteria, while at the same time ensuring that the growth of beneficial bacteria is not hindered. This helps to maintain a healthy balance of both beneficial and pathogenic bacteria within the organism, allowing it to function normally. Food ingredients and preserved food products can be produced using live microorganisms that meet the necessary requirements. A natural microbiota is restored to the system after the patient receives antibiotic medication, which has the effect of restoring its beneficial effects [259]. Furthermore, it functions as an inhibitor to the activity of gut microbiome that have been brought into the body as a result of pollution in the environment and the consumption of incorrect foods.

It has been shown that probiotics can effectively limit the growth of harmful bacteria, such as *Clostridium perfringens* [260], *Campylobacter jejuni* [261], *Salmonella enteritidis* [262], *Escherichia coli* [263], *Shigella* species [264], *Staphylococcus* species [265] and *Yersinia* species, [266] in order that foodborne diseases can be avoided. Several studies have been conducted showing that probiotics improve the digestive process, relieve food allergies [267], decrease Candida infections [268] and reduce tooth decay [269]. The B vitamins (such as B9 and B12) are produced naturally by probiotic bacteria such as *Lactobacillus plantarum* and *Lactobacillus reuteri* as well as *Bifidobacterium adolescentis* and *Bifidobacterium pseudocatenulatum*. The bacteria feed on the carbohydrates in the food we consume, and in the process, they produce B vitamins. These vitamins are then absorbed into the bloodstream and used by the body for various metabolic processes. In addition to these benefits, they also enhance the absorption of minerals and vitamins, boost immune system effectiveness, and increase organic acids production, as well as increase amino acids production [270,271]. A probiotic-induced immunological stimulation has been observed in the form of increased immunoglobulin secretion, an increase in macrophage and lymphocyte activity, as well as an increase in interferon production. It has been suggested that probiotics can possibly have an impact on the congenital and acquired immune systems through their effects on their metabolites, chemical composition of cellular walls and DNA [258]. Probiotics have a number of health advantages, but some research revealed several problems with them, such as unidentified molecular mechanisms, strain-specific behavior, the difference in responses of short-lived, autochthonous, and allochthonic microorganisms, antibiotic resistance, horizontal gene transfer and maintenance of vitality and stability during shelf life [272]. Furthermore, live probiotics appear to be impacted by host-specific factors in the gastrointestinal system, which activate bacterial genes involved in the production and degradation of nutrients [273].

### 6.3. Prebiotics

In addition to probiotics, prebiotics can be used in addition to or in substitution of probiotics as additional supplements [256]. The development of bio-therapeutic formulations that contain the right strains of bacteria and the right prebiotics, both of which work together to promote the growth of probiotics in the colon and small intestine, is believed to be a method to increase the probiotic effect. A number of studies have been carried out in which these “enhanced” probiotic formulations have been found to be significantly more effective in stimulating and protecting the immune system than their component components alone [274,275]. Foods that contain prebiotics may have a number of health benefits, including positively affecting gut health. Several studies have shown that those who consume vegetables and other fruits on a regular basis are less likely to develop bowel cancer as compared to those who do not. Several factors contribute to this action, including inulin and oligofructose [276]. In addition to their many benefits, such prebiotics have been shown to reduce high blood levels of low-density lipoprotein, boost the immune system, increase calcium absorption, support normal gut pH levels, have a low calorie value, and ease symptoms such as peptic ulcers and vaginal mycosis [277]. Other impacts of inulin and oligofructose on public health are the prevention of tumorigenesis, the prevention of lactose intolerance and the prevention of tooth decay [278].

Osmotic properties can lead to adverse effects of prebiotics due to their high dietary fiber, which can draw water into the digestive tract and cause bloating and gas, as well as constipation. Prebiotics’ adverse effects are strongly affected by the length of their chain. A prebiotic containing a shorter chain length, however, may have a greater adverse effect [279].

### 6.4. Synbiotics

**Figure 4:**
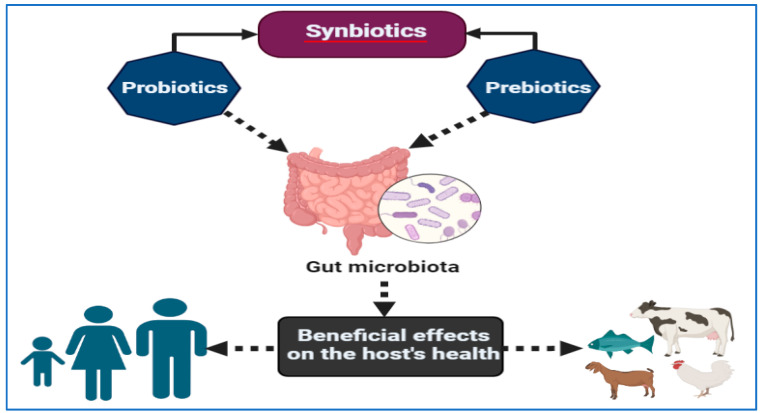
In synergy synbiotics, probiotics and prebiotics work together to improve the health of the gut microbiome—benefiting both humans and animals.

Synbiotics are food ingredients or nutritional supplements that combine probiotics and prebiotics in a synergistic manner [280]. There are a variety of synbiotic products that have a variety of functions, including stimulating the growth of certain native strains of bacteria that already exist in the intestinal mucosal lining and enhancing the survival of beneficial microbes introduced into feed or food [281,282]. However, it is important to keep in mind that the effect of synbiotics on improving health may be determined by the unique balance of probiotics and prebiotics that each individual possesses [283]. Despite the wide range of combinations that can potentially be used to control gut microbiota in humans, using synbiotics for the control of microbial population appears to have a positive outcome [283]. By improving the establishment and/or persistence of the companion bacteria in combination with probiotics or prebiotics, synergistic synbiotics may benefit clinical results more than probiotics or prebiotics taken alone. A properly constructed synergistic synbiotic can likewise prove to be an effective way to turn non-responders into responders, and therefore, foster the efficacy of therapy across a wider client base while enhancing therapy success rates. As shown in Figure 4, a synergistic synbiotic is a combination of probiotics and prebiotics that work together to improve the health of the gut microbiome [284]. This can lead to improved digestion, better nutrient absorption, and reduced inflammation, all of which can help the body better respond to therapies. Some studies performed on older adults revealed that synbiotics dramatically reduced cardiovascular risk factors, metabolic syndrome prevalence and insulin resistance markers [285].

### 6.5. Phytobiotics

A phytobiotic is an element that is non-nutritive and has antibacterial, anti-inflammatory and transcriptional modulating properties [52]. As phytobiotics contain a large number of pharmacologically active chemicals, they have been suggested as a promising therapeutic option [286]. Phytocompounds have the ability to bind free radicals, inhibit xenobiotic activation enzymes and activate detoxication enzymes. These compounds have antiviral, antimicrobial and immunomodeling properties, in addition to being able to stimulate appetite and act as flavors [287]. A study by Micciche et al. [288] evaluated both pre- and post-harvest effects of plant-derived essential oils against *Campylobacter* in poultry production chains, including carvacrol, thymol and cinnamaldehyde. *Campylobacter jejuni* counts were reduced by 2.4–4 logs by washing broiler chickens’ skin with a 2% carvacrol suspension, as this essential oil has been proposed as an antibacterial wash treatment for postharvest poultry. Likewise, lactic acid, citric acid and citrus extract have been shown to be effective against *Escherichia coli* O157 in vitro and in a model rumen system, and this cocktail may be useful in controlling this pathogen in animals [289].

### 6.6. Postbiotics

The term postbiotics refers to preparations of inanimate microorganisms and their components that have been proven to benefit host health [290]. Several microbiota reside in the human intestine, and postbiotic substances may contribute to the maintenance of this microbiome [291]. With the premise that human health should not depend on bacterial survival, postbiotics have emerged as a potential way for addressing the possible dangers of live cell probiotics [292]. A great deal of interest has been shown in using postbiotics in farm animals instead of antibiotics. Due to the bans on antibiotics in many countries, alternative methods to provide antimicrobial effects have gained popularity [293]. The antibacterial properties of postbiotics appear to be one of their benefits [294], although the research on them is still in its early stages. Studies have revealed that postbiotic substances possess antibacterial (pathogenic and spoiler bacteria) properties, which prevent infectious diseases from spreading and food from spoiling. Consequently, these chemicals prevent pathogenic bacteria from colonizing the intestine, thereby preventing disorders of the intestine such as leaky gut syndrome [295]. Among the factors that affect postbiotic antibacterial capabilities are the type of target microbe, the concentration of postbiotics and the nature of the prebiotic source [293].

Postbiotics affect the body’s microbial flora and prevent invading bacteria from replacing the existing gut bacteria, producing antibacterial compounds and lowering ambient acidity [39]. The rate of intestinal infections and other gastrointestinal disorders is dramatically reduced when intestinal microbiota balance is preserved and fermentation and production of post-antibiotics are established [296,297]. Through their attachment to bacterial receptors, postbiotics prevent pathogenic microorganisms, such as toxin-producing *Clostridia* and *Esherichia coli*, from colonizing the gut lumen. In most countries, including Europe, *Bacillus* strains have largely been used to produce postbiotics over the last few decades [298]. Among the most important products of postbiotics are short-chain volatile fatty acids and exopolysaccharides, which are synthesized in the laboratory [299]. A variety of bacterial and fungal strains produce postbiotics in fermented foods, including yogurt, sauerkraut, pickled vegetables and kombucha [300,301]. They are most commonly found in *Lactobacillus*, *Bifidobacterium*, *Streptococcus*, *Eubacterium*, *Faecalibacterium* and *Saccharomyces*.

### 6.7. Development of Vaccines and Immunotherapies

The use of antibodies as therapeutics on humans and animals to ensure passive immunity for the prevention of pathogen colonization through antibodies generated in biological units is currently in development [302]. The development of vaccines against prominent foodborne bacteria is providing a boost to the efforts at the moment [303]. A Japanese pharmaceutical company, Takeda, is performing phase II clinical trials on a potential norovirus vaccine, after researchers established its efficacy against numerous prevalent viral strains [304]. A consortium to produce vaccines against *Shigella* and enterotoxigenic *Escherichia coli* has been funded with USD $50 billion by the Bill & Melinda Gates Foundation since 2007. Vaccinations, according to studies, result in quick, cost-effective results and establish immunity in an individual or population [305], but sanitation programs are more difficult to implement broadly and take longer to achieve substantial gains [306]. A health economist at the non-profit organization PATH says vaccines can supplement water and food cleanliness changes. Various levels of research are underway to develop vaccines against diseases of the bowel (or “enteric”). A number of companies are working on norovirus, and Takeda is one of them. Takeda’s vaccine is one of the most advanced available. Norovirus is the cause of gastroenteritis, a diarrhoeal disease that kills approximately one million people annually. According to WHO, 35,000 of those deaths occurred as a result of norovirus, which spreads via food.

There are already a few vaccines available to prevent foodborne illnesses. As a result of an evaluation of evidence indicating that rotavirus vaccination can provide up to 90% protection against the disease in the years following vaccination, WHO added a rotavirus vaccine to its list of recommended vaccines in 2009. Approximately 453,000 children died from rotavirus worldwide in 2008, according to WHO [303]. It is relatively straightforward to produce vaccines for foodborne illnesses for bacteria that impact both affluent and developing countries. A norovirus vaccine, for example, is projected to prevent approximately 1 million to 2.2 million cases of illness in the United States every year, according to the U.S. Centers for Disease Control and Prevention. In accordance with government estimates, if the vaccination is effective for two years, up to $2.1 billion could be saved in treatment expenditures [307]. In diarrheal endemic countries, only a few licensed vaccinations against various intestinal pathogens are available [308]. Many formulations are in clinical or preclinical development, but none can provide long-term protection, which requires frequent boosters despite efforts to increase safety, immunogenicity, and efficacy. Although several trials are currently carried out, vaccines against foodborne pathogens are difficult to develop because the pathogens can vary significantly in their genetic makeup, making it difficult to create a vaccine that is effective against all strains of a particular pathogen. Additionally, there is a risk of creating a vaccine that could make people sick, so additional safety measures need to be taken into account. We therefore need to collect information on vaccines’ safety, tolerability, immunogenicity and protective efficacy in many countries owing to financial, social and political constraints.

## 7. Conclusions

There has been a growing demand in recent decades for methods that determine the identity of foodborne pathogens due to the rise in consumption of freshly prepared food and food products with a relatively short shelf life. In order to address food safety and public health challenges, numerous technologies have been built for identifying foodborne pathogens. There is a growing amount of commercial viability for early screening techniques that have been developed over the past few years. Despite the fact that conventional methods for identifying foodborne pathogens are time-consuming, labor-intensive and rely on complex culturing techniques, they are still effective and considered the gold standard methods applicable to the majority of cases. To address the drawbacks of conventional detection techniques, some rapid detection technologies have been developed over the past few decades, including PCR, biosensors and MALDI-TOF MS. The ability to quickly detect foodborne pathogens in food products is vital to preventing the occurrence of foodborne illnesses and for preventing their spread throughout the community—and moreover, swift action is required to prevent this occurrence. There are many ways in which communicable diseases can spread from one person to another, among which food products are a primary option. Therefore, it is imperative to seek out techniques that can guarantee consumer protection in an efficient, safe and simple manner and which can be adapted by a wide range of consumers. It is clear from its characteristics that bacteriophages have properties that make them particularly suitable for antibacterial strategies, particularly in the field of food hygiene. Numerous studies have demonstrated that phages, probiotics, prebiotics, synbiotics, phytobiotics and postbiotics are extremely effective in eradicating a wide range of foodborne pathogens and that they are not toxic to humans. In the food industry, for example, producers are concerned about the growth of bacteria that are resistant to antibiotics, as well as the growth of biofilms that are extremely durable. Food products do not suffer from a reduction in quality as a result of phage application. There is still a considerable amount to be done to ensure that phage preparations are widely used in food safety, even though phage-based solutions have made steady progress. Amid the shortcomings of the presently used antibacterial treatments, novel approaches are highly desired. A phage product that can be applied is certainly an option worth considering. The development of vaccines against foodborne pathogens is currently underway, providing protection against multiple pathogens.

## Data Availability

Not applicable.
